# A synthetic building operation dataset

**DOI:** 10.1038/s41597-021-00989-6

**Published:** 2021-08-10

**Authors:** Han Li, Zhe Wang, Tianzhen Hong

**Affiliations:** Lawrence Berkeley National Laboratory, Building Technology and Urban Systems Division, Berkeley, 94720 United States

**Keywords:** Energy modelling, Energy and behaviour, Civil engineering

## Abstract

This paper presents a synthetic building operation dataset which includes HVAC, lighting, miscellaneous electric loads (MELs) system operating conditions, occupant counts, environmental parameters, end-use and whole-building energy consumptions at 10-minute intervals. The data is created with 1395 annual simulations using the U.S. DOE detailed medium-sized reference office building, and 30 years’ historical weather data in three typical climates including Miami, San Francisco, and Chicago. Three energy efficiency levels of the building and systems are considered. Assumptions regarding occupant movements, occupants’ diverse temperature preferences, lighting, and MELs are adopted to reflect realistic building operations. A semantic building metadata schema - BRICK, is used to store the building metadata. The dataset is saved in a 1.2 TB of compressed HDF5 file. This dataset can be used in various applications, including building energy and load shape benchmarking, energy model calibration, evaluation of occupant and weather variability and their influences on building performance, algorithm development and testing for thermal and energy load prediction, model predictive control, policy development for reinforcement learning based building controls.

## Abstract (summary)

**Table:** 

Measurement(s)	energy consumption • power demand • humidity • air flow rate • Occupancy • water flow rate
Technology Type(s)	computational modeling technique
Factor Type(s)	temporal interval
Sample Characteristic - Environment	building

Machine-accessible metadata file describing the reported data: 10.6084/m9.figshare.14682948

## Background & Summary

Building sector accounts for over 30% of the final energy consumption and emit about one-third of the greenhouse gas (GHG) emissions worldwide^1^. Residential and commercial buildings consume about 60% of the electricity globally^2^. Improving building energy efficiency becomes essential to meet energy savings and carbon emission reduction goals^3^. As the electrification progresses, there is an ongoing trend to replace traditional fossil fuel with renewable power generations. European Union has a plan to reach renewable power generation at least 20% of the energy demand by 2020, and 32% by 2030^4^. In the United States, the renewable target is to reach 14% by 2025 and 30% by 2030^5^. The growing penetration of renewables requires buildings to be flexible so that the supply and demand can be balanced. Under this circumstance, Grid-Interactive Efficient Buildings (GEB) has become a hot research topic in recent years^6^. Improving buildings’ energy efficiency and flexibility while maintaining good quality of building services and indoor environmental quality is of core interest in the building science domain.

Building energy models provide critical support to researches aiming for the aforementioned goals. In general, the models can be classified into (1) physics-based (white-box) models, which simulate the building physics with detailed building and system characteristics and operation schedules; (2) reduced order (grey-box) models, which represent building physics with simplified equations identified with building operation data or by human expertise; (3) data-driven (black-box) models, which utilize contextual, environmental, or energy features with statistical or machine learning techniques to predict future energy and/or environmental trends in buildings. Those models have been used in different phases of building lifecycles. For example, physics-based whole building energy simulations have been widely used in the building design phase to assist building energy code compliance^7,8^. Predictive building controls using physics-based models^9^, grey-box models^10^, and data-driven models^11^ are proposed and implemented during the operation phase. Those models are also widely used for fault detection and diagnostics in the operation phase^12^.

Regardless of the modeling approaches, a comprehensive building operation dataset is valuable. For the physics-based models, the system-level or end-use level information, and the time-series data can help improve model assumptions and calibrations. For grey-box and data-driven approaches, such a comprehensive dataset is critical for training reliable models. As of now, there are numerous efforts in either collecting data from measurements^13–16^ or synthesizing data with simulations^17,18^. However, each of the dataset has its strengths and limitations. For instance, the Building Data Genome Project 2 dataset is a collection of whole building electrical, heating and cooling, water, steam and solar meters, and on-site weather data for over 1,600 non-residential buildings^13^. However, it doesn’t provide more granular information about the system and thermal zones. CU-BEMS provides system-level sub-metering of electricity consumptions, and zone-level indoor environmental measurements^14^. But it doesn’t contain system-level operation data and data only spans for over a year. Other common limitations of the existing datasets include the lack of clear metadata that describes the building systems and meter structures, and occupancy information at high spatial and temporal resolutions. Therefore, there remains a gap of a comprehensive set of building operation data. In this paper, we present AlphaBuilding - a synthetic building operation dataset^19^ created using recently developed modeling techniques. The uniqueness of this dataset includes:

- Simulated with reference building energy models with detailed thermal zoning in EnergyPlus, a physics-based building performance simulation engine
- Stochastic occupancy schedules are used in simulations to represent occupancy diversity and dynamics at the space level
- Dynamic lighting, MELs, and HVAC system operation schedules are used in simulations
- Real weather data for three typical climate locations in 30 years are used in simulations to capture the yearly variations of building performance due to weather variability
- BRICK schema^20^ is used to create a metadata model for the building, system equipment, sensors and meters which ensures interoperability of the dataset
- HDF file format is used to store the resources (OpenStudio models, weather files) and data (metadata, time-series data) to facilitate big data analytics and high-performance computing

In the rest of the paper, we introduce the method used to generate the dataset, the data records structure, an exploration of the data and comparison with some public building dataset, and the example use cases of the developed dataset.

## Methods

In this section, we describe the method used to create the synthetic building operation dataset, which includes: an overview of the workflow, building and model information, key modeling assumptions, and the simulation implementations.

### Overall workflow

**Fig. 1:**
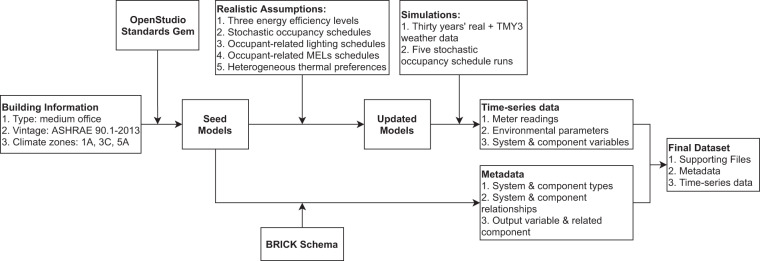
Overall workflow of the simulation data generation.

The overall workflow to generate the synthetic building operation dataset is shown in Fig. 1. The process starts with the basic building information input, including the building type, vintage, and climate zones. We used OpenStudio Standards Gem^21^, a Ruby library of the OpenStudio Software Development Kit (SDK), to create the seed models. Then, we modified the seed models to represent three energy efficiency levels by changing the building envelope properties, lighting, MELs, and HVAC system efficiencies. We then modified the schedules for zone-level occupancy, lighting, MELs, and thermostat setpoint, to reflect more realistic building operations^17^. Next, we ran simulations with the updated models, which utilized thirty years’ historical weather data plus a Typical Meteorological Year (TMY3)^22^ weather data. For each weather file, we ran five times of the stochastic occupancy simulation to update the occupancy and related assumptions. More details about the building, modeling assumptions, and simulations will be presented in the next section. The simulations yielded time-series data including the whole-building and end-use energy metering, indoor and outdoor environmental parameters, and system and component variables (e.g., zone thermostat setpoints, VAV terminal supply air temperature). We converted the original CSV-format data into HDF5 format to improve the read and write efficiency and reduce the data’s disk size requirements. In the meantime, we created a metadata model using the BRICK schema for the building model, which describes the type, quantity, and relationships among the key system and components in the building. Finally, the metadata and the time-series data constitute the complete dataset.

The synthetic data generation framework could be reused to create new datasets with different assumptions (e.g., building type, vintage, weather condition, system efficiency, occupant behavior). We open-source the code of this framework, which allows readers to reproduce this dataset or generate custom datasets. Details about the source code and guidance on how to generate this dataset or other custom datasets are explained in the Code availability section.

### Modeling assumptions

#### Building

**Fig. 2:**
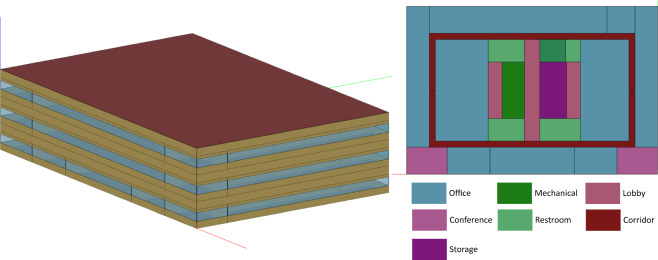
Building geometry and thermal zones.

The U.S. Department of Energy (DOE) developed a suite of reference commercial building models^23^ which represent 70% of the commercial buildings in the U.S., and have been used in a variety of research and applications. The model we used is a medium-sized office building with three floors and a total of 52,628 square feet (4,890 square meter) floor areas. We used the detailed version of the reference model which has more space types and more granular zoning than the original one. The 3D visualization and the zoning configuration are shown in Fig. 2. The building consists of 12 space types - open and enclosed office rooms, conference room, classroom, dining area, lobby, corridor, stair, storage, restroom, plenum, and mechanical room.

#### Climate & weather

To reflect different climates and weather conditions’ impacts on building operations, we considered three locations corresponding to three climate zones - Miami (1 A, hot and humid), San Francisco (3 C, moderate/mild), and Chicago (5 A, cold winter and hot summer). For each location, we used thirty-year historical weather data and one TMY3 weather data.

#### Efficiency level

Many factors including the physical properties of building envelope, age and efficiency of building systems can influence the overall building performance. We considered three energy efficiency levels by modifying the component and system characteristics. The key assumptions regarding the efficiency levels are summarized in Table 1.

**Table 1:** Assumptions for three system and envelop efficiency levels.

Efficiency Level	Low	Standard (ASHRAE 90.1–2013)	High
COP of AHU	1.8	2.4	3
water heater thermal efficiency	46.7%	62.3%	77.9%
gas burner efficiency	48.0%	64.0%	80.0%
VAV reheat coil efficiency	60.0%	80.0%	95.0%
fan total efficiency	36.3%	48.4%	60.5%
pump motor efficiency	18.0%	24.0%	30.0%
envelope thermal resistance	0.75 standard level	varies by climate	1.25 standard level

#### Systems

The building is served with three packaged variable air volume (PVAV) air handling units (AHUs), with each one serving one floor. The AHUs are equipped with air-cooled direct expansion (DX) cooling coil and gas heating coil. Each zone is served by a VAV terminal unit with electric reheat coils. Properties of the lighting and MELs systems are shown in Table 2.

**Table 2:** Lighting, MELs and Occupancy information of the main space types.

Space Type	lighting power density (W/m2)	MELs power density (W/m2)	occupancy density (m2/person)
open office	13.2	12.9	12.2
enclosed office	14.9	11.7	20
conference room	16.5	13.5	2.5
classroom	16.7	12.5	2.7
corridor	8.9	3.9	n.a.
stair	9.3	n.a.	n.a.
dining room	8.7	13.4	9.3
lobby	12.1	3.6	9.3
mechanical room	12.8	3.6	n.a.

### Occupant-related variabilities

**Fig. 3:**
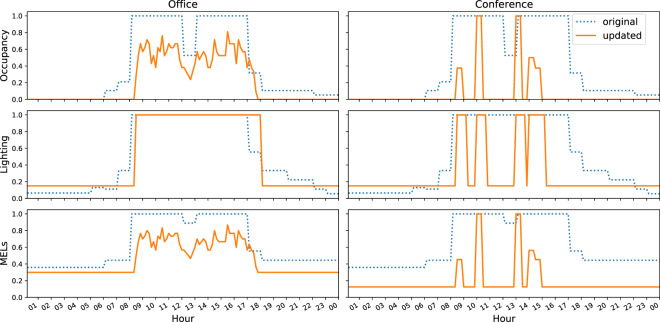
Original and updated schedules comparison for an open office and a conference room.

Traditionally, building energy simulation uses homogeneous and static occupant schedules, which lack temporal and spatial variations. However, a variety of studies have shown that occupants’ movements in office buildings are dynamic and stochastic^24,25^. Other literature also revealed the correlation between occupancy and the operation of lighting^26,27^, and MELs^28–30^. In addition, occupants tend to have different thermal preferences due to their age, gender, and cultural differences^31^. Therefore, we considered the dynamic occupancy and its correlation with lighting and MELs operations, as well as occupants’ diverse thermal preferences. The dynamic occupancy schedules were generated with an agent-based stochastic occupancy simulator^24^. Figure 3 shows the comparison between the original and updated occupancy, lighting, and MELs schedules in an open office and a conference room.

To address the limitation of traditional building energy simulations where most thermal zones are assumed to have unifying heating and cooling thermostat setpoint temperatures, we adopted non-unifying heating and cooling setpoints (identified as two normal distributions^32^), which are inferred from the ASHRAE Global Thermal Comfort Database II^33^. Table 3 shows the comparison between the original and updated setpoint and setback temperatures.

**Table 3:** Original and updated thermostat setpoint and setback temperatures.

	Original	Updated
Heating	Setpoint: 21.1 °C	Setpoint: N(22.8, 1.872) °C
	Setback: 15.6 °C	Setback: 15.6 °C
Cooling	Setpoint: 23.9 °C	Setpoint: N(23.7, 1.192) °C
	Setback: 29.4 °C	Setback: 29.4 °C

The correlated lighting and MELs operating schedules, and the diverse thermostat setpoints are generated with an OpenStudio extension Gem (library)^34^. Since each stochastic occupancy simulation yields a unique schedule, we conducted variability simulation for each location, efficiency level, and weather file. Therefore, we have 3 (locations) x 31 (weather files) x 3 (efficiency levels) x 5 (occupancy and internal heat gains variability simulations) = 1,395 unique models.

## Data Records

**Fig. 4:**
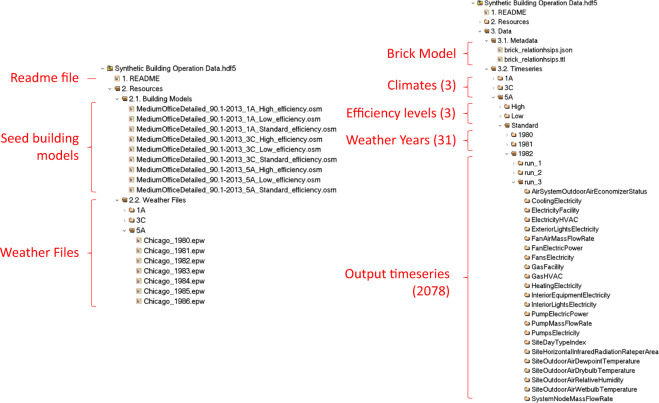
Dataset file structure.

As illustrated in Fig. 1, the final dataset^19^ is composed of the metadata and the time-series operation data. We used HDF5^35^ - a hierarchical data format to store the whole dataset. The HDF5 format supports fast extraction and slicing of large datasets in a hierarchical way. The datasets are organized as groups and encoded with UTF8 for standard electronic communications. The total file size is about 1.2 TB (about 2.5 TB in CSV format). Figure 4 show the structure of the data file.

### Metadata

**Fig. 5:**
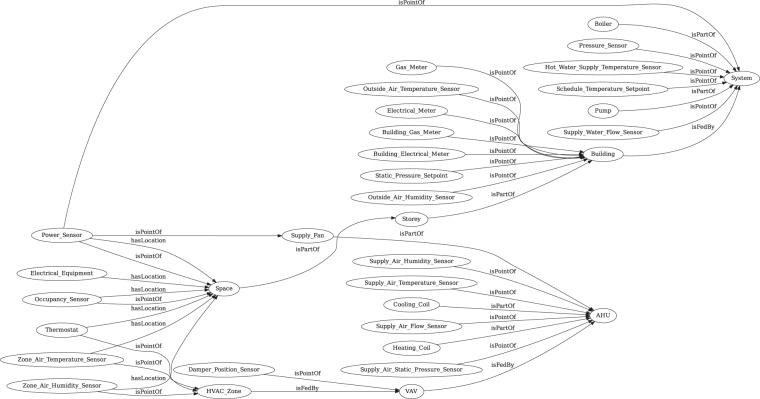
Brick schema of the model.

Metadata is critical to building operation analytics because it provides semantic information about the physical, spatial, and virtual assets and their relationships in buildings. We used Brick schema to store the metadata of the building models. Brick is an open-sourced data schema aimed to provide standardized semantic descriptions for building assets. Since the physical component of the models in all 1,395 simulations are the same, a single Brick model needs to be created. The Brick model is represented with the Resource Description Framework (RDF) language^36^ in the Turtle (TTL) file format. The RDF language is a general-purpose language which can be written in a compact and natural text form. Figure 5 shows entity classes in the building and their relationships generated by the Brick TTL Viewer (https://viewer.brickschema.org/). Each entity class may have multiple instances. For example, the “VAV” class has a relationship of “isFedBy” with the “AHU” class. In the building model, there can be multiple “VAV” instances fed by the same “AHU” instance. The detailed relationships of the specific instances can be found in the metadata TTL file.

### Time-series data

Time-series data is the major component of the dataset. All the variables in the dataset were reported at a 10-minute interval for a whole year. For each of the 1,395 simulations, 35 CSV tables are generated with each consisting of 1 to 377 timeseries. We converted the CSV tables into HDF5 data tables and saved them in the master file. Depending on the types and numbers of variables, the size of a single file ranges from less than 2 Megabyte (MB) to around 150 MB. Table 4 summarizes the hierarchy and naming conventions of the time-series data files, the variable types and dimensions and the approximate size of each file. The <path_identifier> specifies the climate, efficiency level, weather file, and stochastic occupancy simulation runs. For instance, “…/3 C/Standard/TMY3/run_4/ZoneElectricEquipmentElectricPower” contains the simulation results of zone-level electric equipment power demand of the building with standard efficiency level in climate zone 3 C, using the TMY3 weather file, with the forth stochastic simulation results as the occupant-related variability schedules. The units of variables can be found in the header name of each file. The total size of the time-series data generated from a single run is about 890 MB.

**Table 4:** Time-series data file summary.

Name	Variable Type	Dimension (rows columns)	Size (MB)
<path_identifier>/AirSystemOutdoorAirEconomizerStatus	System Variable	52560 3	3
<path_identifier>/CoolingElectricity	Energy	52560 1	2
<path_identifier>/ElectricityFacility	Energy	52560 1	2
<path_identifier>/ElectricityHVAC	Energy	52560 1	2
<path_identifier>/ExteriorLightsElectricity	Energy	52560 1	2
<path_identifier>/FanAirMassFlowRate	System Variable	52560 3	3
<path_identifier>/FanElectricPower	Power	52560 3	3
<path_identifier>/FansElectricity	Energy	52560 1	2
<path_identifier>/GasFacility	Energy	52560 1	2
<path_identifier>/GasHVAC	Energy	52560 1	2
<path_identifier>/HeatingElectricity	Energy	52560 1	2
<path_identifier>/InteriorEquipmentElectricity	Energy	52560 1	2
<path_identifier>/InteriorLightsElectricity	Energy	52560 1	2
<path_identifier>/PumpElectricPower	Power	52560 1	2
<path_identifier>/PumpMassFlowRate	System Variable	52560 1	2
<path_identifier>/PumpsElectricity	Energy	52560 1	2
<path_identifier>/SiteDayTypeIndex	Other Variable	52560 1	2
<path_identifier>/SiteHorizontalInfraredRadiationRateperArea	Other Variable	52560 1	2
<path_identifier>/SiteOutdoorAirDewpointTemperature	Other Variable	52560 1	2
<path_identifier>/SiteOutdoorAirDrybulbTemperature	Other Variable	52560 1	2
<path_identifier>/SiteOutdoorAirRelativeHumidity	Other Variable	52560 1	2
<path_identifier>/SiteOutdoorAirWetbulbTemperature	Other Variable	52560 1	2
<path_identifier>/SystemNodeMassFlowRate	System Variable	52560 377	153
<path_identifier>/SystemNodePressure	System Variable	52560 377	153
<path_identifier>/SystemNodeRelativeHumidity	System Variable	52560 377	153
<path_identifier>/SystemNodeTemperature	System Variable	52560 377	153
<path_identifier>/ZoneAirRelativeHumidity	Zone Variable	52560 68	29
<path_identifier>/ZoneAirTerminalVAVDamperPosition	Zone Variable	52560 65	28
<path_identifier>/ZoneElectricEquipmentElectricPower	Power	52560 47	20
<path_identifier>/ZoneLightsElectricPower	Power	52560 65	28
<path_identifier>/ZoneMeanAirTemperature	Zone Variable	52560 68	29
<path_identifier>/ZoneMechanicalVentilationMassFlowRate	Zone Variable	52560 65	28
<path_identifier>/ZonePeopleOccupantCount	Zone Variable	52560 28	13
<path_identifier>/ZoneThermostatCoolingSetpointTemperature	Zone Variable	52560 68	29
<path_identifier>/ZoneThermostatHeatingSetpointTemperature	Zone Variable	52560 68	29

## Technical Validation

In this section, we explored the synthetic building operation data to illustrate the data coverage and quality. We then compared the simulated energy consumptions with several public datasets - the building performance database and a group of office buildings in California.

### Explorations

The time-series data of this dataset include indoor and outdoor environmental parameters, system operational variables, zone-level parameters, and energy and power demand. The explorations aim to illustrate the patterns of those time-series data at a high level, as well as the variabilities due to historical weather conditions and stochastic occupancy and occupant-related system operations in the simulations.

#### Historical weather

**Fig. 6:**
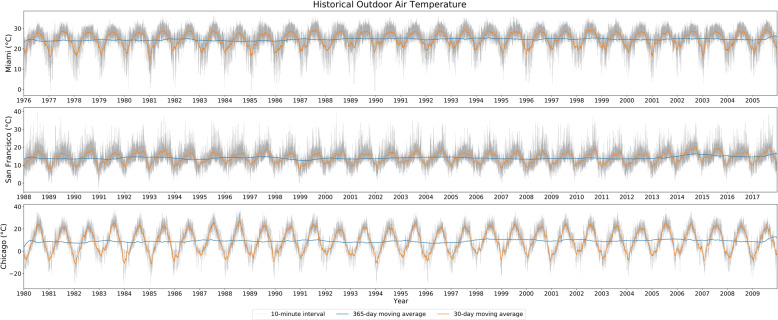
Historical weather data trends.

Figure 6 shows the historical weather conditions in three aforementioned locations in 10-minute intervals, 30-day moving average, and 365-day moving average trends, respectively. The weather data for Miami ranges from the year of 1976 to 2005, which has an average outdoor air temperature of about 24 degree Celsius. The weather data for San Francisco are between 1988 and 2017, with an average outdoor air temperature of about 15 degree Celsius. And the weather data for Chicago are between 1980 to 2009, with an average outdoor air temperature of around 8 degree Celsius.

#### Zone occupant count

**Fig. 7:**
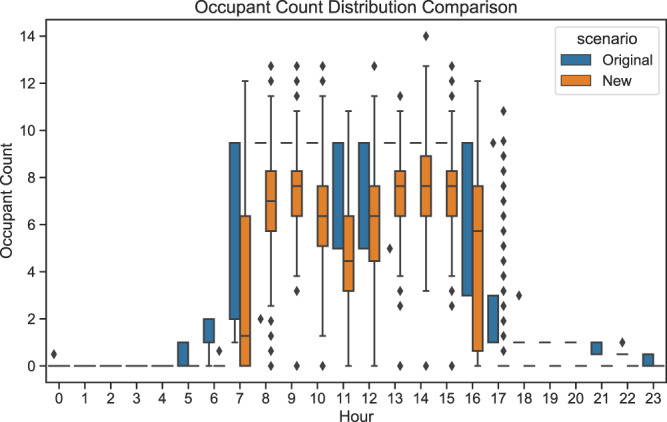
Occupant count distribution comparison an open-plan office.

Figure 7 shows the comparison of the hourly occupant count distributions of an open-plan office with the original and proposed occupancy schedule, respectively, in a whole year. It can be seen that for most of the operating hours, the original scenario has almost fixed occupancy in the entire year. While the new scenario has variable occupancy schedules.

#### Zone thermostat setpoints

**Fig. 8:**
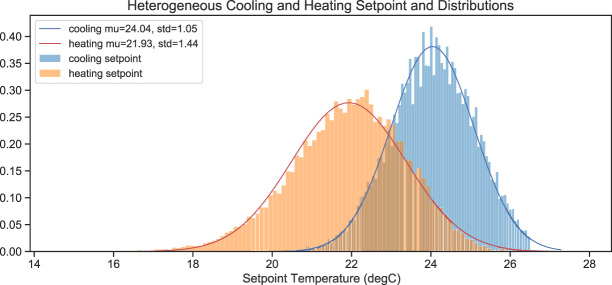
Zone thermostat setpoint distributions.

Figure 8 shows the heterogeneous zone thermostat setpoint distributions from the simulation results.

#### Zone air temperature

**Fig. 9:**
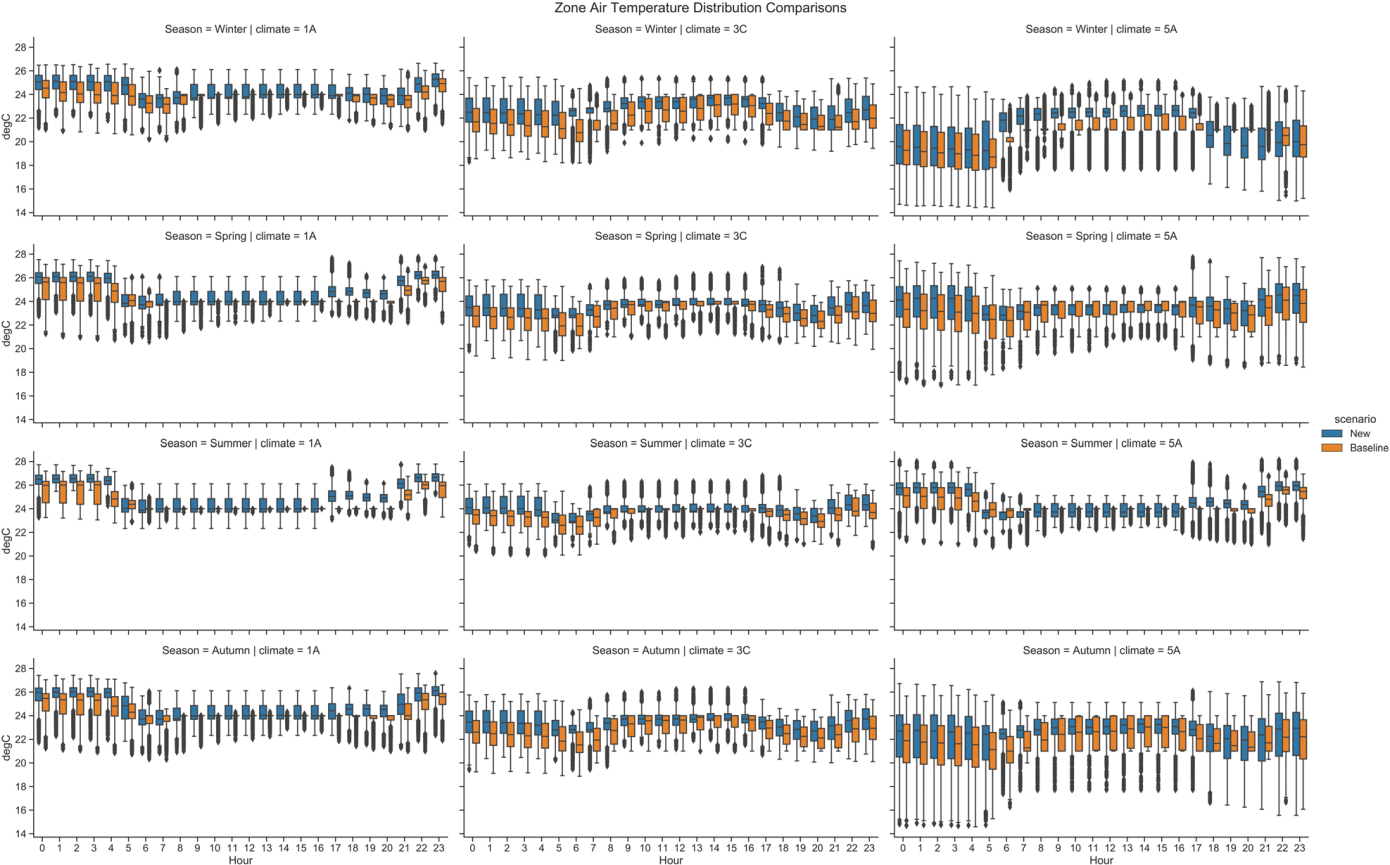
Zone temperature distributions - workdays.

As the result of the heterogeneous zone thermostat setpoints, the simulated zone air temperature also has more higher variabilities compared to the original scenario where all the thermal zones have the same thermostat setpoint schedules. Figure 9 shows the comparison of the zone air temperature distribution between the original and new scenarios across all the office zones in the model. The results are derived from the simulation with TMY3 weather file and are broken down to different climates and seasons. It can be seen that for the climate 1 A (Miami), the new scenario has wider temperature distributions in all seasons in the working hours (8am to 18 pm). In climate 5 A (Chicago), the new scenario has wider temperature distributions in winter and summer in the working hours. Those hours have the most needs for space conditioning. Thus, the heterogeneous thermostat setpoints have more obvious impacts on the zone air temperature distributions. While for climate 3 C (San Francisco), the original and new scenario have comparable ranges or even the original scenario has a wider distribution. The reason is San Francisco has a mild climate, thus the zone air temperature is less regulated by the HVAC system.

#### Energy consumptions

**Fig. 10:**
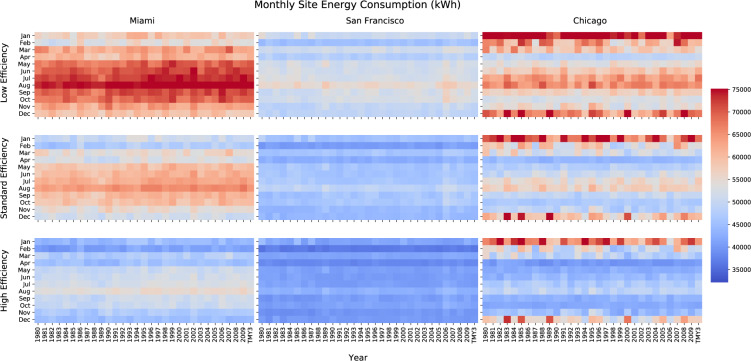
Whole-building energy consumption heatmaps.

The monthly whole-building energy consumption heat maps are created to visualize the impact of historical weather and efficiency levels on energy consumption. Figure 10 shows the heatmaps in three locations (from left to right) and three efficiency levels (from top to bottom). For each subplot, the horizontal axis shows the year and the vertical axis shows the month of a year. The heat maps show consistent trends in all three locations - as the energy efficiency level goes up, the energy consumptions are reduced. In terms of different locations, the order of energy consumption is: Miami > Chicago > San Francisco. That’s because Miami is cooling-dominant, Chicago has cold winters and relatively hot summers, while San Francisco has a mild climate which results in less heating and cooling demands in buildings.

**Fig. 11:**
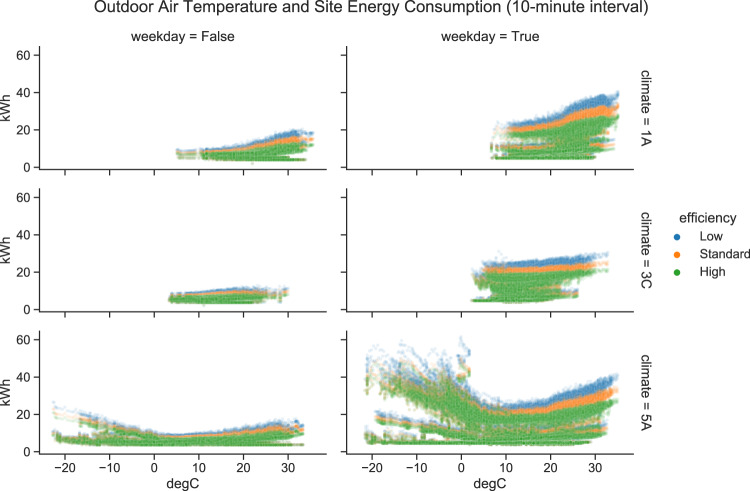
Outdoor air temperature and site energy consumption relationships.

Figure 11 further reveals the impacts of weather and energy efficiency levels on the building energy consumption. The figure shows the relationship between outdoor air temperature and the site electricity consumption, organized by day types and climates, and color-coded by the energy efficiency levels. Intuitively, the weekend consumption is lower than weekday consumption for all three climates. In addition, as the energy efficiency increases, the electricity consumption reduces. The weekday trends show distinct patterns in the three climates. In climate 1 A, the electricity consumption remains relatively constant when the outside temperature is below 20 °C, and starts to rise as the temperature goes up. In climate 3 C, the trends are similar except for a gentler slope when the energy consumption starts to rise as the temperature increases from about 17 °C. For climate 5 A, we can see some scatters are always at low kWh level regardless of the outside temperature change. Those are the baseloads that are not weather-sensitive, such as lighting, MELs, and other essential electricity consumptions. For other scatters, we observe a heating-sensitive trend when the outside temperature is below 5 °C, a cooling-sensitive trend when the outside temperature is above 20 °C, and a relative flat trend in between.

**Fig. 12:**
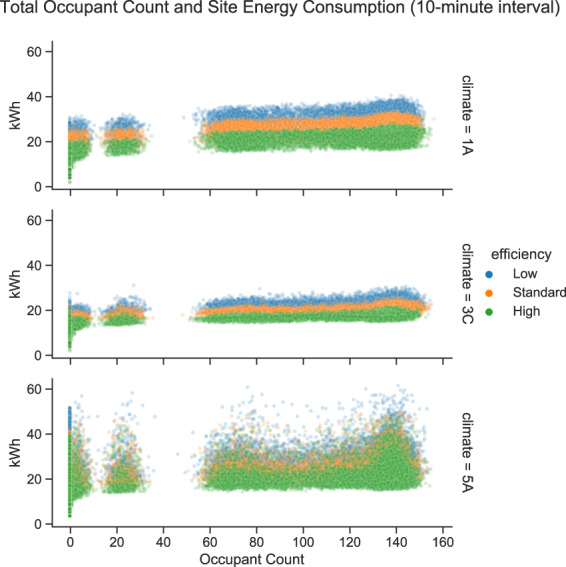
Total occupant count and site electricity consumption relationships.

Figure 12 shows the relationship between the total occupant count and the site energy consumption. The plot is broken into climate zones and color-coded by the energy efficiency level. Again, as the efficiency level increases, the site energy consumption decreases. Moreover, we observe a slightly positive correlation between the number of occupants and the site energy consumption. However, the coefficient of determination (R-squared) value of the correlation varies among three locations, as indicated by the sparseness of the scatters. In climate 3 C where there is less heating and cooling demand, non-HVAC systems energy consumption has a higher proportion in the total energy consumptions. Since the non-HVAC system (i.e., lighting and MELs) power demands are positively correlated with the number of occupants, the correlation between the site energy consumption and occupant count is more significant. On the contrary, in climate 1 A and 5 A where the space conditioning demands are higher, HVAC systems consume a bigger portion of the energy. Because the dynamic thermostat setpoint schedules are not determined by the number of people, the correlations between the occupant count and the site energy consumption are less significant.

### Comparison with public datasets

#### Annual Site EUI comparison with BPD

**Fig. 13:**
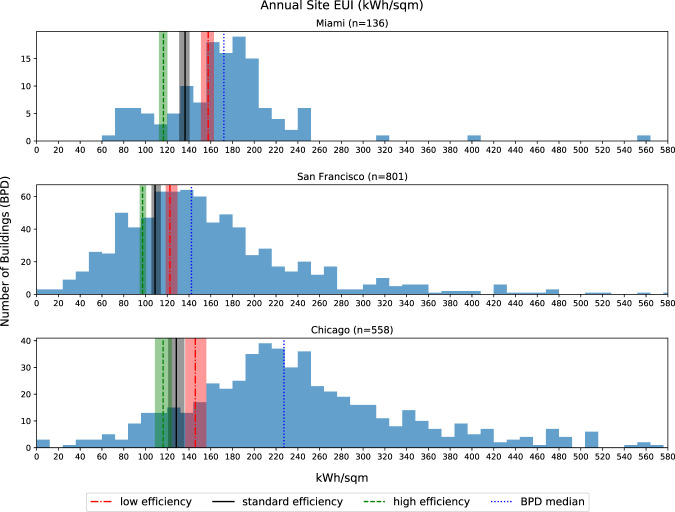
Annual Site Energy Use Intensity Comparison with BPD.

The building performance database (BPD)^37^ is the largest publicly-available source of measured building energy performance data in the United States. It contains information about building type, location, physical and operational characteristics for over 1 million commercial and residential buildings. The comparison between the synthetic dataset with BPD is at annual level because the energy performance data in BPD is aggregated to annual intervals. Figure 13 shows the annual site energy use intensity (EUI) comparisons in three locations simulated. In each subplot of Fig. 13, the blue histogram shows the distribution of the annual site EUIs from real buildings in BPD, and the blue vertical line represents the median building EUI in BPD. The red, black, and green vertical lines indicate the low, standard, and high energy efficiency levels, respectively. The colored bands around the three vertical lines show the ranges of the annual site EUIs, which are the results of different weather data and stochastic occupancy simulations. In all three locations, the simulated EUIs are lower than the BPD medians. The main reason is that the buildings in the synthetic dataset follow ASHRAE 90.1–2013 standard requirements. However, the buildings from BPD have older vintages, which are less energy efficient. We also found that the ranges of the simulated EUIs vary by location. For example, the EUI ranges of buildings in Miami and Chicago are wider than the ranges in San Francisco. The reason is that Miami and Chicago have more hot and cold days which results in higher variabilities in HVAC energy consumptions.

#### Load Profile comparison with PG&E dataset

**Fig. 14:**
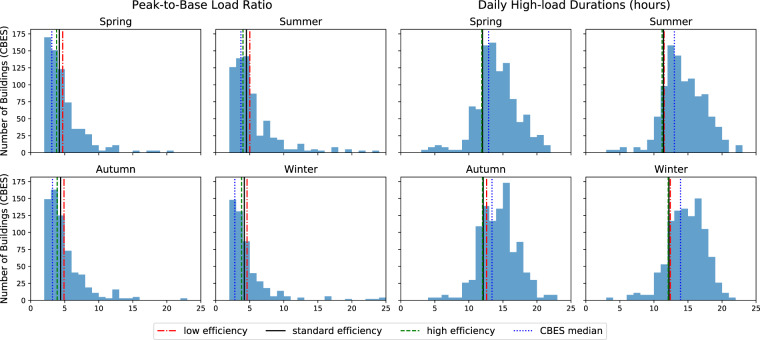
Load Shape Benchmarking with CBES.

To evaluate the daily load profile patterns, we compared the synthetic load profiles with a group of buildings. The dataset contains over 400 small- to medium-size office buildings which are served by Pacific Gas & Electricity (PG&E) customers in California. Therefore, we only selected the synthetic load profiles of buildings in San Francisco for the comparisons. We used a web-based application - Commercial Building Energy Saver (CBES)^38^, to benchmark two key load profile parameters in different seasons - high-load durations and peak-to-base ratio, shown in Fig. 14. The definitions of those two parameters can be found in this paper^39^. The high-load durations are the number of hours in a day when the building’s electrical load is at high level, which usually overlaps with the operation hours. In all four seasons, the average high-load durations of the synthetic load profiles are between 12 to 13 hours, whereas the PG&E medians are slightly higher - around 13 to 14 hours. This means our operation duration in the simulations are on the lower side in the building operation schedules. The peak-to-base ratio is the average ratio of power demand in high-load hours (i.e. operation hours) to it in the low-load hours (i.e. off-hours). It can be seen that in all four seasons, the peak-to-base ratio of the synthetic load profiles are higher than the distribution medians. And as the energy efficiency level improves, the peak-to-base ratio decreases, which means the buildings with high efficiency assumptions are even more energy efficient in off-hours.

## Usage Notes

The dataset is in HDF5 format and the total size is about 1.2TB. We recommend users to process the dataset with programming languages like Python, which has libraries that provide easy HDF file read/write capabilities. A more detailed note about the dataset and a Jupyter notebook with Python script to explore the dataset are available at the dataset’s GitHub page (https://lbnl-eta.github.io/AlphaBuilding-SyntheticDataset). The libraries used for the data processing are included in the Jupyter notebook. For users who want to view the file without heavy data manipulations, publicly available free software programs like HDF® VIEW are recommended.

## Data Availability

A step-by-step guidance and the source-code to generate this dataset, and a notebook to explore and visualize the data can be found at the dataset’s GitHub page (https://lbnl-eta.github.io/AlphaBuilding-SyntheticDataset).
